# A temperature-controlled mattress cover is associated with improved subjective sleep in elite male Australian-rules football athletes: A 14-night parallel-group field study

**DOI:** 10.1080/23328940.2026.2703946

**Published:** 2026-07-30

**Authors:** Shauna Stevenson, Yazmin Hayes, Haresh T. Suppiah, Matthew W. Driller

**Affiliations:** School of Allied Health, Human Services, and Sport, La Trobe University, Melbourne, Australia

**Keywords:** Thermoregulation, sleep quality, sleep technology, thermal comfort, sleep environment, bedding

## Abstract

Sleep is recognized as a critical factor in athletic recovery and performance. However, elite athletes often experience inadequate sleep, and research suggests that disturbances in thermoregulatory processes may be a contributing factor. This study aimed to investigate the effects of a temperature-controlled mattress cover on thermal comfort and sleep outcomes in professional athletes. Thirty-two elite male Australian Rules Football athletes (mean ± SD age: 23 ± 4 years) participated in a 14-night intervention using a temperature-controlled mattress cover (Eight Sleep Pod 3) (POD, *n* = 16) or their own mattress (CON, *n* = 16). The Brief-Pittsburgh Sleep Quality Index (B-PSQI) was used to analyze changes in sleep quality. Post-intervention, the POD group rated perceived thermal comfort, sleep impact, satisfaction, and recovery on a 1–10 Likert scale. Linear mixed models showed significant group ×time interaction favoring POD over CON for total sleep time (TST; +29 min, *g* = 1.41, *p =* 0.008), improvements in sleep quality (−1.0, *g* = -3.06, *p* < 0.001), and global score (−2.4, *g* = -2.43, *p* < 0.001) all taken from the B-PSQI. Perceived thermal comfort in the POD group did not change significantly from pre- to post-intervention (median [Q1-Q3]l 3.0 [2.0–4.0] and 4.0 [2.0–4.0], *p* > 0.05). The use of a temperature-controlled mattress cover was associated with improvements in subjective sleep measures in our cohort of athletes. These preliminary findings suggest that the device may be a promising noninvasive strategy for improving perceived sleep quality, pending confirmation in randomized studies with validated objective sleep measures.

## Introduction

Sleep quality is considered essential for effective recovery in athletic populations [1,2]. Thermoregulation, the physiological mechanism for maintaining a stable core temperature, plays a fundamental role in sleep quality [3]. Prior to sleep onset, increases in peripheral heat dissipation elevate distal skin temperature [4] and contribute to a decline in core body temperature (T_core_), a thermoregulatory process closely coupled with circadian rhythms and associated with faster sleep onset [5]. T_core_ continues to decline during the initial phases of sleep, promoting sleep consolidation [6]. Optimal sleep is associated with skin temperatures between 33.5–35.5°C [3], with deviations potentially disrupting sleep architecture and increasing awakenings [5]. Emerging sleep technologies actively regulate bed temperature and monitor sleep-related data, offering personalized thermal environments [7]. However, evidence of their effectiveness, especially in athletes, remains limited.

Athletes have problems obtaining the recommended 7–9 hours of sleep per night, despite sleep being considered one of the most effective strategies for recovery [8]. Elite athletes frequently experience insufficient or disrupted sleep due to a range of physiological, psychological, and environmental stressors [9] including suboptimal sleep environments that may impair thermoregulation [5]. Further, evening exercise elevates T_core_, heart rate, and sympathetic activity, which can delay sleep onset and its associated reduction in core temperature, ultimately disrupting sleep continuity [6]. Yet, there are limited studies in elite athletes assessing sleeping systems that could influence their sleep quality and recovery, highlighting a gap in assessing thermoregulatory influences like temperature and bedding systems [5].

Exposure to thermally uncomfortable environments, whether excessively warm or cold, has been associated with decreased total sleep time, prolonged sleep onset latency, and increased wakefulness in young adults [10]. These effects are attributed to the body’s heightened thermal sensitivity during sleep, which relies on stable ambient and microenvironmental temperatures for sleep continuity [11]. Inadequate bedroom environments, particularly those outside the optimal ambient range of 18–22°C, have been identified as significant contributors to disturbed sleep [12]. Impaired heat dissipation under such conditions can hinder the initiation and maintenance of sleep [13] highlighting the importance of thermal supportive sleep environments.

Advancements in sleep technology have led to bedding systems that dynamically regulate bed temperature to support thermoregulation during sleep. Temperature-controlled mattress covers (e.g. Eight Sleep Pod) use active heating and cooling mechanisms to modulate the sleep surface per user preferences [14]. In healthy adults, such technologies have demonstrated improvements in deep sleep duration, reduced resting heart rate, and enhanced perceived sleep quality [15,16]. In a controlled trial, temperature-controlled mattress covers resulted in significantly increased odds ratio (OR) of subjective total sleep time (OR = 0.36), sleep quality (OR = 0.12), reduced sleep latency (OR = 0.23), reduced sleep heart rate (−1.5 bpm), and improved heart rate variability (+4.1 ms) in the general population [7]. More recently, a randomized crossover study reported significant improvements in perceived outcomes, including calmness of sleep, ease of falling asleep and waking, feeling refreshed, sleep satisfaction and thermal comfort following the use of temperature-controlled bedding in healthy adults [17]. While these findings are promising, further research is needed to validate these benefits, particularly among athletic populations.

Therefore, the current study aimed to investigate the effect of a temperature-controlled mattress cover compared with a standard mattress on perceived sleep outcomes in elite Australian Rules Football (AFL) athletes over 14 nights. Additionally, biometric measures, perceived thermal comfort, sleep satisfaction, and recovery were examined among athletes who used the temperature-controlled mattress cover.

## Materials and methods

Thirty-two elite male (mean ± SD age: 23 ± 4 years) AFL players (classified as Tier 4 elite level athletes using the athlete classification framework by McKay [18]) participated in the current study during the AFL pre-season. Exclusion criteria included those under 18 years old, use of sleep-influencing medications or supplements, diagnosed sleep disorders, or incompatible bed sizing. Informed consent was obtained, and ethical approval was granted by the institutional Human Research Ethics Committee (HEC24002).

Data collection occurred between December and March in Melbourne, Australia (coordinates = 37.8136°S, 144.9631° E; Southern Hemisphere summer and early autumn). According to Bureau of Meteorology records for Melbourne, monthly mean daily maximum temperatures over this period ranged from approximately 24–27°C, and monthly mean daily minimum temperatures (typically reached overnight) ranged from approximately 13–17°C, with conditions across the 2024–25 summer running marginally warmer than the long-term seasonal average for Greater Melbourne (Bureau of Meteorology, 2025). Indoor bedroom temperatures in Australian homes during this period were typically warmer than outdoor overnight minima due to building retention. Data collection took place during the pre-season phase of the AFL schedule. Participants completed approximately five days of training per week, comprising 8–12 sessions including on-field (~4 sessions) and strength and conditioning training (~4 sessions). Total training volume was approximately 18 hours per week.

### Study design

**Figure 1.**
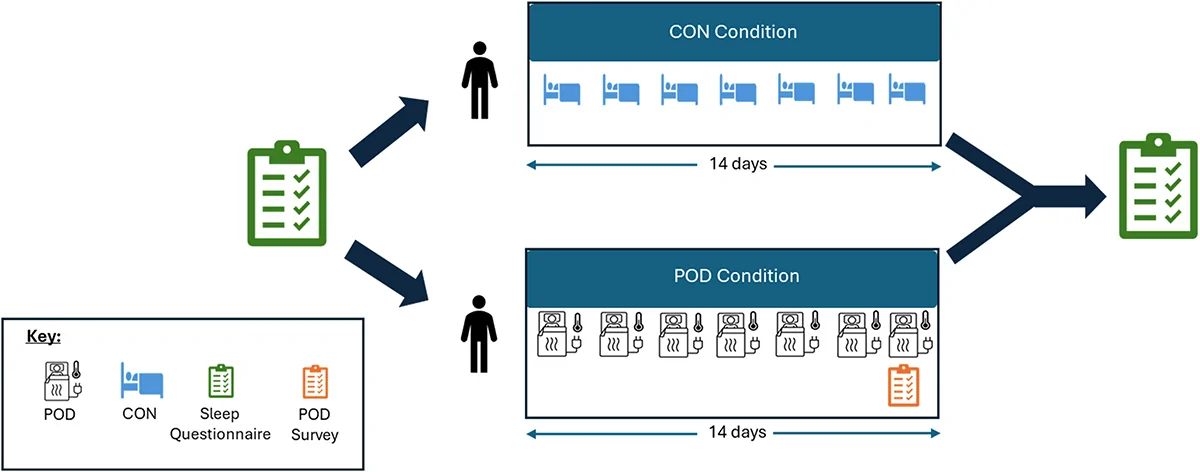
Schematic of the study protocol. This study employed a two-group, between-participant design. Participants were assigned to either a temperature-controlled mattress cover condition (POD) or a control group sleeping on a standard mattress (CON) (*n* = 16 per group). Both groups completed a sleep quality questionnaire before (pre) and after (post) the two-week intervention period. The POD group completed a survey at the end of the intervention period.

The current study implemented a parallel-group, between-subject experimental design with repeated measures (see Figure 1). Participants were allocated to either a temperature-controlled mattress cover group (POD) or a control group (CON) for two weeks. Participant allocation was non-random and determined pragmatically based on device availability. Participants in the POD group slept on a temperature-controlled mattress cover (Eight Sleep, Pod 3, New York, NY, USA) for a minimum of 14 nights (mean = 19 nights, range 14–24 nights). Participants in the CON group slept on their usual standard mattress cover, which did not include temperature control (mean = 14 nights). Participants completed sleep quality questionnaires pre- and post-intervention. Participants in the POD group also completed thermal comfort questionnaires pre- and post-intervention.

### Temperature-controlled mattress cover

**Figure 2.**
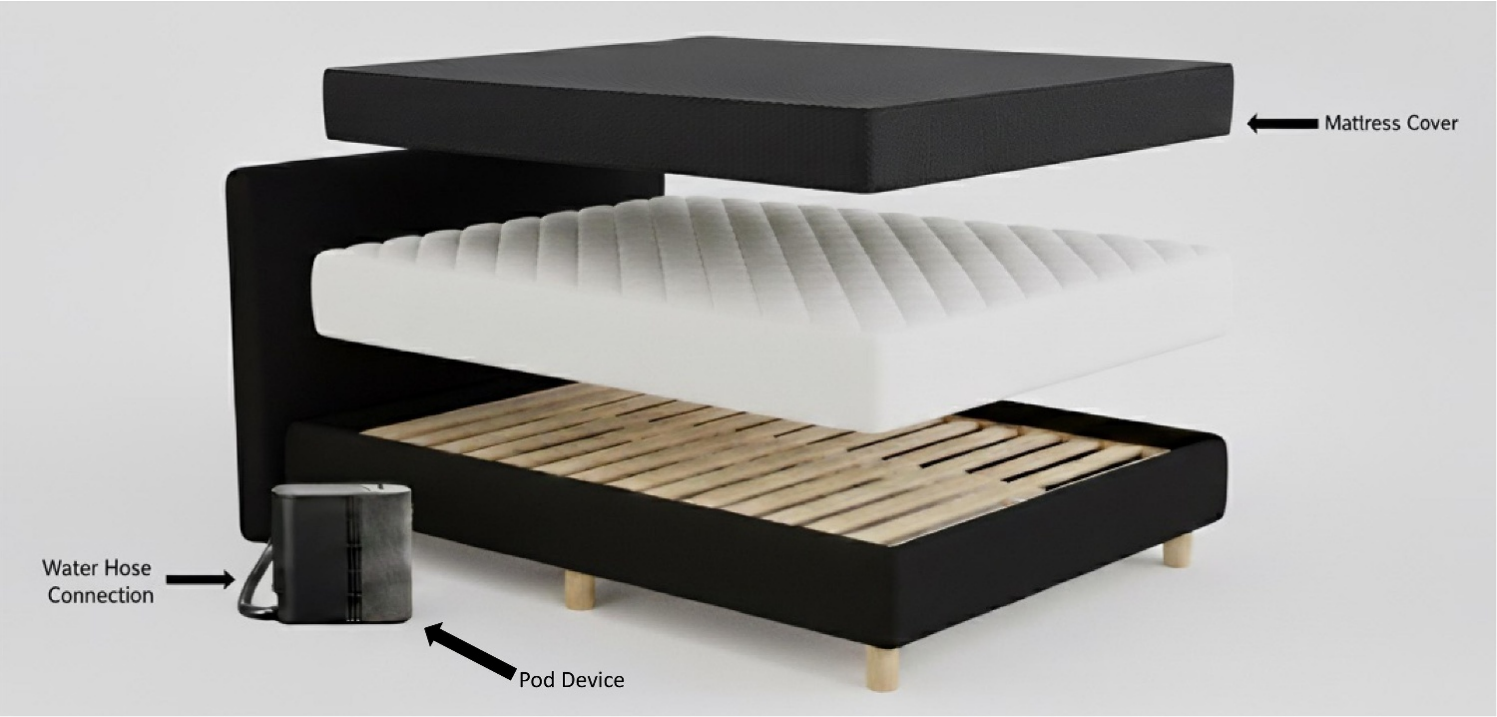
The temperature-controlled mattress cover and Pod device used in the current study. The system consists of a mattress cover connected via a water hose to the external Pod device, which regulates the bed temperature.

The Pod (Figure 2) adjusts bed temperature via water tubes embedded in the mattress cover, controlled through a smartphone app. The Pod captures biometric data overnight via ballistocardiographic and load-cell sensors embedded in the mattress cover, which are processed by the manufacturer’s proprietary algorithm to derive sleep architecture variables (total sleep time, total time in bed, sleep latency, sleep efficiency, sleep stages), and biometric metrics (heart rate, HRV, respiratory rate). Biometric metrics have been internally validated by the manufacturer; however, to the author’s knowledge, these outputs have not yet been independently validated in the peer-reviewed literature. Accordingly, these data are reported descriptively only and were not used in inferential analyses. Participants could customize temperature settings across three phases: Bedtime (until 15 minutes of consistent sleep), Early (first 4 hours), and Late (until wake), within a ~ 13–43°C range. Although general guidelines exist [7], participants were free to adjust settings.

### Perceived outcomes

Questionnaires were administered via REDCap (Research Electronic Data Capture, version 15.0.26), pre- and post-intervention. The Brief-Pittsburgh Sleep Quality Index (B-PSQI) assesses self-reported sleep patterns and quality with the wording amended to reflect on sleep over the past two weeks rather than the last month, in order to align with the 14-day data collection period used in each condition of the current study. Otherwise, item content and scoring of the B-PSQI was unchanged. Questions included bedtime, wake time, sleep latency (SOL), sleep disturbances, sleep quality (SQ), total time in bed (TTiB) (i.e. the participant’s reported time spent in bed regardless of whether they were asleep), and total sleep time (TST) (i.e. time spent asleep within the TTiB), with global scores ranging from 0–15, whereby higher scores (>5) indicate poorer sleep quality [19]. Thermal comfort was rated on a 5-point Likert scale (1 = very uncomfortable, 2 = uncomfortable, 3 = neutral, 4 = comfortable, and 5 = very comfortable).

Using 10-point scales, participants in the POD group rated their satisfaction with the device (1 = “Did not enjoy it and would not use again,” 5 = “Neutral/no strong feelings” and 10 = “Loved it and would use again”), the impact on sleep (1 = “Impaired my sleep,” 5 = “No impact on my sleep” and 10 = “Improved my sleep”), and the recovery support (1 = “No support at all,” 5 = “Neutral/no noticeable effect,” and 10 = “Significant improvement on my recovery”) post-intervention. For the B-PSQI, lower scores indicate better perceived sleep, with global scores > 5 typically interpreted as poor sleep quality. In contrast, for the perceptual rating scales (thermal comfort, satisfaction, sleep impact, recovery impact), higher scores indicate more favorable perception.

### Statistical analysis

Statistics were performed in Jamovi (Version 2.6.25, Jamovi, 2018), with statistical significance set at *p* < 0.05. The results are presented as mean ± standard deviations (SD). B-PSQI subscales and global scores were tested for normality using the Shapiro – Wilk test (α = 0.05). As the data were not normally distributed, a two-tailed Wilcoxon signed-rank test was conducted on thermal comfort data collected pre- and post-intervention in the POD group.

Linear mixed model (LMM) analysis was used to evaluate the differences in subjective sleep outcomes between the POD and CON groups. The analysis included fixed factors: group (POD or CON) and time (pre and post). Participants were included as a random effect for all LMM analyses. Total time in bed (TTiB) was calculated as the interval between self-reported bedtimes and waketimes from the B-PSQI, and was included as a covariate in the LMM to account for differences in sleep opportunity, allowing variations in sleep outcomes (e.g. sleep efficiency, TST) to be interpreted independently of time spent in bed. Statistical significance for fixed effects was determined using F tests with the degrees of freedom for F statistics computed using the Satterhwaite approximation method. Assumptions of normality and homoscedasticity were assessed using model residuals. Standardized mean difference (SMD) was computed as Hedge’s *g*. Effect size thresholds were: <0.2 *trivial*, 0.2–0.49 *small*, 0.5–0.79 *moderate*, ≥0.8 *large*. Effect size magnitudes should be interpreted in the context of study design, including the small, non-randomized sample and subjective outcome measures. If the 95% CI spanned both −0.20 and +0.20 (i.e. included values consistent with a small benefit and a small harm), the effect was deemed *unclear*. To assess the robustness of the findings to baseline imbalances between groups, a sensitivity analysis was conducted using an ANCOVA approach, adjusting for baseline values of each outcome.

## Results

Descriptive statistics of Brief-Pittsburgh Sleep Quality Index (B-PSQI) metrics and thermal comfort are displayed in Table 1. Perceived thermal comfort in the POD group did not change significantly from pre- to post-intervention (*p* = 0.562, *g* = 0.14, *trivial*).

**Table 1.** Descriptive statistics for all B-PSQI metrics pre- and post-intervention in the CON and POD groups, presented as mean ± standard deviation of normally distributed variables and median (Q1-Q3) for non-normally distributed variables (thermal comfort).

	CON		POD	
	PRE (Mean ± SD)	POST (Mean ± SD)	PRE (Mean ± SD)	POST (Mean ± SD)
Bedtime (hh:mm)	22:08 ± 0:30	22:03 ± 0:32	22:06 ± 0:46	21:54 ± 0:49
Waketime (hh:mm)	6:33 ± 0:19	6:32 ± 0:15	7:06 ± 0:28	7:15 ± 0:28
Sleep Latency (min)	17 ± 9	15 ± 6	21 ± 10	17 ± 8
Total Sleep Time (h:min)	7 h 21 min ±23 min	7 h 22 min ±31 min	7 h 40 min ±33 min	8 h 17 min ± 40 min
Sleep Efficiency (%)	88.42 ± 4.20	87.44 ± 6.25	85.50 ± 4.99	87.34 ± 8.36
Sleep Disturbances (/5)	1.4 ± 1.0	1.3 ± 0.9	2.1 ± 1.1	1.3 ± 1.0
Sleep Quality (/5)	0.8 ± 0.4	0.9 ± 0.3	1.6 ± 0.6	0.6 ± 0.5
B-PSQI Global Score (/15)	3.1 ± 1.5	3.0 ± 1.6	4.9 ± 2.0	2.6 ± 1.8
Thermal Comfort (/5)	–	–	3.0 (2.0–4.0)	4.0 (2.0–4.0)

**Figure 3.**
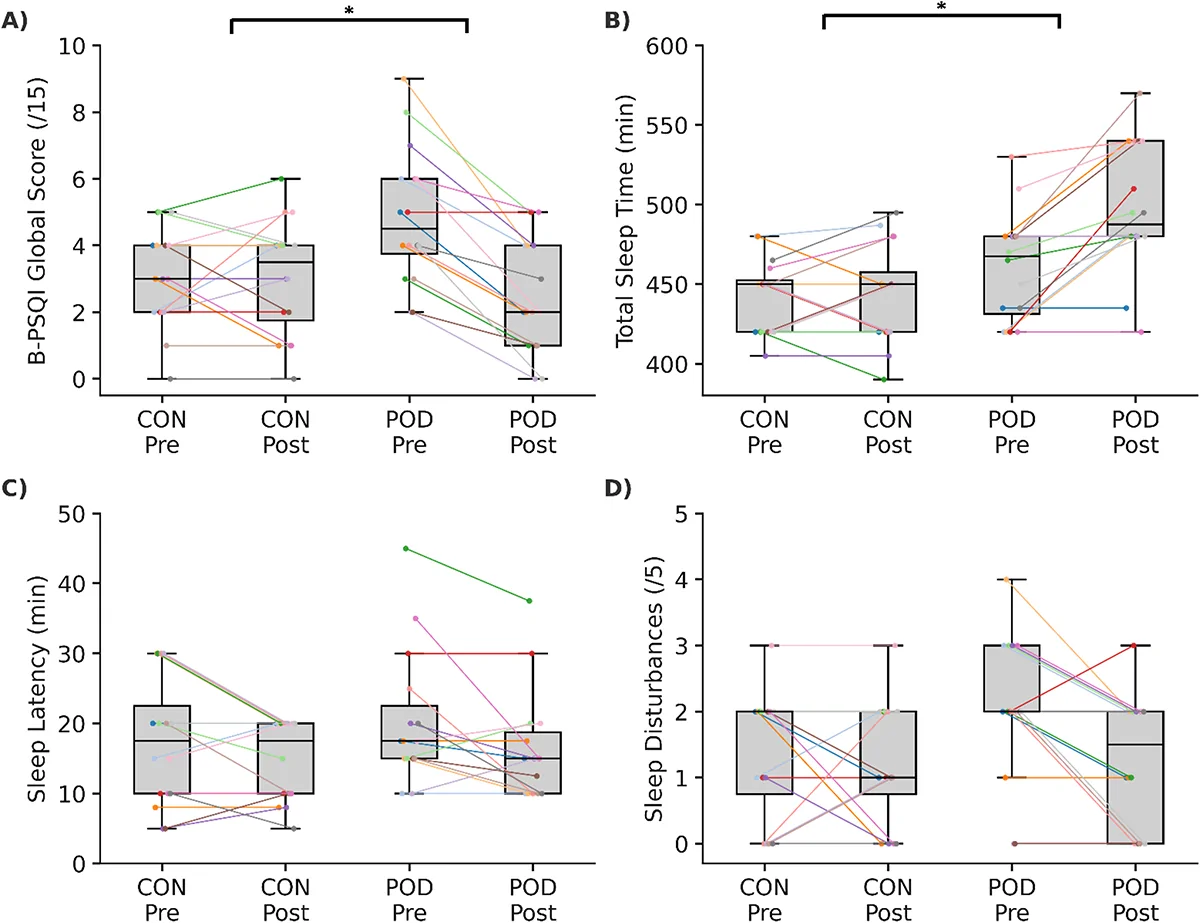
Box plots showing the changes in subjective sleep outcomes A) B-PSQI global score, B) total sleep time, C) sleep latency, and D) sleep disturbances before (pre) and after (post) the intervention period for each group (*n* = 16 per group). Each box spans the 25th–75th percentiles (interquartile range), the central line marks the median, and whiskers extend to 1.5 × IQR. Colored lines and circles show individual participants. *Indicates a significant time x group interaction (*p* < 0.05).

Linear mixed-model (LMM) results for all B-PSQI metrics (subscales and global score) are presented in Tables 2 and 3, with corresponding box-and-whisker plots of key metrics shown in Figure 3. A significant Group ×Time interaction was found for TST (F (1, 27) = 8.117, *p* = 0.008) with a *large* effect (*g* = 1.41), whereby only the POD group showed an increase in TST from pre-to post (mean difference = + 29 min, *p =* 0.003), with little change observed in the control condition (mean difference = +1 min, *p* > 0.05). Further, the POD group reported significantly longer TST post-intervention (mean difference = +43 min, *p* = 0.01). Furthermore, a significant Group ×Time interaction was found for sleep quality (F (1, 31) = 38.066, *p* < 0.001) with a *very large* effect (*g* = −3.06), whereby the POD group reported significantly poorer sleep quality than the control group at baseline (pre-intervention) (mean difference = −0.78, *p* < 0.001), but demonstrated a meaningful improvement in sleep quality from pre- to post-intervention (mean difference = +0.92, *p* < 0.001). Lastly, a significant Group × Time interaction was reported for B-PSQI global score (F (1, 30) = 23.804, *p* < 0.001) with a *very large* effect (*g* = −2.43), whereby the POD group demonstrated a large reduction (indicating better overall sleep quality) from pre- to post-intervention (mean difference = −2.48, *p* < 0.001). Post-hoc analysis revealed no further significant Group ×Time interaction findings on remaining subjective sleep outcomes.

**Table 2.** Linear mixed-model interaction, including the estimate, *p*-value, and standardized mean difference (SMD) for the following subjective perceived outcomes: sleep onset latency (SOL) (min), total sleep time (TST) (min), and sleep efficiency (SE) (%), assessed pre- and post-intervention in athletes who slept on a temperature-controlled mattress cover (POD group) and those who used a standard mattress (CON group).

Predictor	Estimate (B) (95% CI)	*p-*value	Effect Size (Hedge’s *g*)	Estimate (B) (95% CI)		*p-*value	Effect Size (Hedge’s *g*)		Estimate (B) (95% CI)	*p-*value	Effect Size (Hedge’s *g*)
	**SOL (min)**				**TST (h:min)**			**SE (%)**			
Intercept	17.3 (14.6–20.0)			7:40 (7:29–7:50)					87.3 (85.7–88.9)		
Time (Pre/Post)	−3.6 (−6.0–1.1)	**0.007**	−0.51 *Moderate*	0:15 (0:05–0:26)		**0.008**	0.72 *Moderate*		1.7 (−0.8–4.2)	0.190	0.35 *Unclear*
Group (POD-CON)	1.6 (−4.–7.6)	0.603	0.23 *Unclear*	0:28 (0:05–0:51)		**0.018**	1.37 *Large*		2.4 (−1.4–6.2)	0.215	0.49 *Unclear*
Total Time in Bed	1.2 (−2.5–4.8)	0.529	0.17 *Unclear*	0:10 (−0:04–0:25)		0.167	−0.50 *Unclear*		−5.1 (−7.9–2.4)	**<0.001**	−1.06 *Large*
Time*Group	−1.6 (−6.4–3.2)	0.503	−0.23 *Unclear*	0:29 (0:09–0:50)		**0.008**	1.41 *Large*		4.5 (−0.4–9.5)	0.079	0.930 *Large*
Random Effects											
Between subjects SD	6.758			0.166					2.882		
Within subjects SD	4.634			0.112					4.749		
ICC	0.680			0.597					0.269

**Table 3.** Linear mixed-model interaction, including the estimate, *p*-value, and standardized mean difference (SMD) for the following subjective perceived outcomes: sleep disturbances (/5), sleep quality (/5) and Brief-Pittsburgh sleep quality Index (B-PSQI) global score, assessed pre- and post-intervention in athletes who slept on a temperature-controlled mattress cover (POD group) and those who used a standard mattress (CON group).

Predictor	Estimate (B) (95% CI)	*p-*value	Effect Size (Hedge’s *g*)	Estimate (B) (95% CI)	*p-*value	Effect Size (Hedge’s *g*)		Estimate (B) (95% CI)	*p-*value	Effect Size (Hedge’s *g*)
	**Sleep Disturbances**			**Sleep Quality**			**B-PSQI Global Score**			
Intercept	1.5 (1.2–1.8)			1.0 (0.8–1.1)				3.4 (2.8–3.9)		
Time (Pre/Post)	−0.5 (−0.8–0.1)	**0.017**	−0.64 *Moderate*	−0.4 (−0.6–0.3)	**<0.001**	−1.32 *Large*		−1.3 (−1.8−0.8)	**<0.001**	−1.32 *Large*
Group (POD-CON)	0.4 (−0.3–1.1)	0.321	0.48 *Unclear*	0.3 (−0.0–0.6)	0.093	0.93 *Unclear*		0.4 (−0.9–1.6)	0.565	0.38 *Unclear*
Total Time In Bed	0.0 (−0.5–0.5)	0.962	0.02 *Unclear*	−0.1 (−0.3–0.2)	0.556	−0.21 *Unclear*		0.4 (−0.3–1.2)	0.233	0.47 *Unclear*
Time*Group	−0.7 (−1.4–0.0)	0.069	−0.93 *Large*	−1.0 (−1.3–0.7)	**<0.001**	−3.06 *Very large*		−2.4 (−3.3−1.4)	**<0.001**	−2.43 *Very large*
Random Effects										
Between subjects SD	0.699			0.371				1.440		
Within subjects SD	0.714			0.313				0.951		
ICC	0.489			0.585				0.696		

For outcomes that did not show a significant Group × Time interaction, the LMM identified a significant main effect of time (pre- to post-intervention) for sleep latency (SOL) (min) (F (1, 30) = 8.288, *p* = 0.007), sleep disturbances (F (1, 32) = 6.345, *p* = 0.017). Main effects of time for TST, sleep quality, and B-PSQI global score are not interpreted here, as the significant Group × Time interactions reported above for these outcomes supersede interpretation of the corresponding main effects. No significant effects of time were reported on sleep efficiency (SE).

Furthermore, the LMM demonstrated a significant group effect on participants’ TST (min) (F (1, 32) = 6.235, *p* = 0.018) whereby TST was longer for participants in the POD group compared to CON. No other significant effects of group were reported on the remaining subjective sleep outcomes, including SOL, SE, sleep disturbances, sleep quality, or B-PSQI global score.

Lastly, the LMM identified a significant effect of total time in bed on SE (F(1, 45) = 14.422, *p* < 0.001), whereby SE was lower for participants who spent longer in bed. No other significant effects of total time in bed were reported on subjective sleep outcomes, including SOL, sleep disturbances, sleep quality, or B-PSQI global score.

Sensitivity analysis adjusting for baseline differences between groups using an ANCOVA approach yielded similar results to the primary LMM analyses, confirming that the findings were robust to baseline imbalances. Specifically, after adjusting for baseline values, the between-group difference (POD vs CON) at post-intervention remained significant for TST (adjusted B = 4.34 min, *p* = 0.058]), sleep quality (adjusted B = 3.501, *p* = 0.038), and B-PSQI global score (adjusted B = 0.405, *p* = 0.005).

Objective sleep outcomes and biometric measures derived from the mattress cover (proprietary metrics that have not yet undergone independent validation), as well as perceptual outcome scores are shown in Table 4. Participants in the POD group reported high satisfaction with the temperature-controlled mattress cover. On 10-point scales, participants rated the perceived impact of the intervention on sleep positively and reported a moderate perceived influence on physical recovery.

**Table 4.** Descriptive statistics of athletes in the POD group (post-intervention), including the mean and standard deviation (SD) of objective sleep and biometric outcomes derived from the temperature-controlled mattress cover (not yet independently validated), and perceptual outcome scores using 5- or 10-point Likert scales.

	Mean ± SD
Sleep Outcomes	
Bedtime (time of day)	23:12 ± 1:49
Waketime (time of day)	7:36 ± 1:19
Total Sleep Time (h:mm)	7:59 ± 0:31
Sleep Latency (min)	36.5 ± 14.3
REM Sleep (h:mm)	1:47 ± 0:13
Deep Sleep (h:mm)	1:26 ± 0:13
Biometric Outcomes	
Heart rate (bpm)	47.0 ± 4.6
Heart rate variability (RMSSD)	82.2 ± 34.7
Breathing rate (breaths per min)	14.3 ± 1.7
Snoring (min)	0:09 ± 0:07
Perceptual Outcomes	
Sleep Impact /10	7.6 ± 1.4
Satisfaction /10	8.1 ± 1.8
Recovery Impact /10	6.7 ± 1.7

Abbreviations: RMSSD (root mean square of successive RR-interval differences.

## Discussion

The present study examined the effects of a temperature-controlled mattress cover on perceived sleep outcomes compared with a control condition in elite male AFL athletes. Overall, our results indicate that when compared to a control group, the use of a temperature-controlled mattress cover was associated with favorable changes in several B-PSQI subscales, including total sleep time, sleep disturbances, sleep quality, and, importantly, the B-PSQI global score in elite athletes.

These findings are consistent with previous research demonstrating the benefits of thermal bed technologies on sleep perception. For instance, Moyen et al. [7] observed significant improvements in subjective TST (OR = 0.36, *p* = 0.003), perceived sleep quality (OR = 0.12, *p* < 0.001) and perceived SOL (OR = 0.23, *p* < 0.001) when using temperature-controlled mattress covers. Similarly, Stevenson et al. [17] reported improvements in perceived sleep quality (*p* < 0.001) with corresponding *large* effect sizes (*d* = 0.92), supporting the current findings of higher reported subjective sleep quality in the POD group from pre- to post-intervention. Other studies have reported enhanced subjective sleep quality when using thermoregulatory mattress systems [20,21]. For example, the use of a high heat capacity mattress topper also resulted in increased subjective sleep quality in young men [16]. Pasquier et al. [14] demonstrated large improvements in perceived sleep quality, though such subjective gains may be influenced by placebo or expectancy effects. Indeed, a meta-analysis reported no significant effects of bedding strategies on objective measures of SOL, SE, or TST in healthy adults [14]. In contrast, the present study identified shorter subjective SOL and longer TST in the POD group following 14 nights of sleep on the temperature-controlled mattress cover, although previous studies have not found corresponding changes in these parameters when assessed objectively [7,17]. Although objective sleep metrics were captured by the temperature-controlled mattress cover, this technology has not yet been independently validated and only utilized in the POD group, therefore group comparisons cannot be made. These discrepancies underscore the need to integrate both validated subjective and objective sleep assessments in athletes to determine whether perceived improvements in sleep quality translate into physiological improvements over longer periods of time.

Improvements in subjective sleep outcomes in the POD group in the current study may be attributed to placebo or Hawthorne effects [22,23], as athletes in the POD group were aware of the condition they were undertaking, were aware they were being assigned to use novel sleep technology and were able to actively adjust or customize their bed temperature via a smartphone application, which may have enhanced perceived comfort and subsequently influence subjective sleep outcomes. For instance, previous research has reported that thermal comfort and sensation positively impact sleep quality [24,25]. However, the inclusion of a parallel control group allowed natural fluctuations in sleep to be distinguished from intervention-specific changes. While placebo or Hawthorne effects cannot be fully ruled out, particularly in athletic populations where blinding may not be feasible, the subjective benefits in the POD group may reflect both genuine changes and expectancy-driven effects. However, as suggested by Neukirch & Colagiuri [25], incorporating objective sleep measures, as well as core body temperature, may help clarify whether perceived improvements reflect genuine physiological benefits in athletes.

Total time in bed is an important factor influencing the current findings across the intervention period. Although sleep quality improved significantly in the POD group, there were minimal changes to subjective SOL and SE, suggesting that the perceived improvement in sleep quality may have been driven primarily by increases in subjective TST. Further, our results indicate that increased total time in bed was associated with reduced sleep efficiency, with a *large* effect size reported. This negative association indicates that longer reported sleep durations may not necessarily reflect more consolidated or restorative sleep [26]. Importantly, no Group × Time interaction was found for sleep efficiency, therefore suggesting this pattern occurred similarly across groups and does not fully explain the intervention effects.

Unlike most existing studies conducted in general populations, the current study focused on elite athletes, who often experience elevated physical demands [9]. Previous research utilizing thermoregulatory bedding systems in the athletic population has produced mixed findings, highlighting the complexity of sleep regulation in this population. For instance, a high-heat capacity mattress topper did not result in any significant improvements in objective sleep outcomes compared to a low-heat capacity mattress topper in elite badminton players [27], with similar findings reported in endurance runners [21]. However, Aloulou et al. [28] observed that a high-heat capacity mattress improved several objective sleep indices, including shorter SOL in elite rugby union athletes following a match. The authors of this study attributed these benefits to enhanced nocturnal heat transfer and improved thermoregulatory efficiency during sleep [28], suggesting that such technologies may be particularly beneficial under conditions of elevated post-exercise body temperature. The observed subjective improvements in the present study, therefore, suggest that thermoregulatory interventions may offer potential recovery benefits for elite athletes, especially when sleep is compromised by thermal or competitive factors. Future research should investigate the effect of a temperature-controlled mattress cover during the in-season period in team sport athletes, particularly under post-match conditions, as sleep disruption and delayed sleep onset are common following evening games [29], potentially due to increased core body temperature following exercise [30]. Moreover, given that nearly 60% of team-sport athletes report lacking a strategy to address sleep disturbances, these findings underscore the potential for thermoregulatory sleep interventions in high-performance sport environments [31].

The subjective benefits to sleep outcomes identified in the current study may be explained by several candidate mechanisms associated with temperature-controlled bedding, including enhanced thermal comfort, improved circadian thermoregulation, and potential cardiovascular pathways. Although enhanced thermal comfort has been previously proposed as a mechanism through which temperature-controlled bedding may influence sleep, thermal comfort did not change significantly in the current study and is therefore unlikely to be the primary mechanism underlying the observed improvements in subjective sleep. For instance, research using the same technology as the present study reported significant reductions in sleeping heart rate (−1.2 bpm, *p* < 0.001) and an increase in heart rate variability (+7%) in healthy adults sleeping on a temperature-controlled mattress cover, suggesting that cardiovascular responses may be acutely sensitive to thermally-controlled bedding [7]. Previous studies have reported lower sleeping HR when high-heat capacity mattresses are used to enhance nocturnal cooling [15,16]. These observations have been proposed to reflect increased vagal activity, as variations in the bed microenvironment may influence warm-sensitive, sleep-promoting neural pathways, which may support a more restorative physiological state during sleep [12,15,16]. However, these mechanisms were not directly assessed in the present study and should be considered hypothesis generating. Future research should incorporate targeted physiological and thermoregulatory measurements such as core body temperature, gold-standard polysomnography and bed temperature profiles to better elucidate how temperature-controlled bedding influences sleep-related perceptions and responses.

Perceived thermal comfort was rated modestly among athletes in the POD group, median (Q1-Q3) ratings of 3.0 (2.0–4.0) pre-intervention and 4.0 (2.0–4.0) post-intervention out of 5; however, this change was not statistically significant and should not be interpreted as an improvement. Previous research has shown that optimizing the sleep environment and bedding materials can enhance thermal comfort [5] and may positively influence sleep [24]. Therefore, a longer intervention to allow athletes to develop their temperature preferences across different stages of the season may yield more robust and sustained effects in both comfort and sleep quality. Athletes assigned to the POD group also reported high satisfaction with the devices’ comfort and usability, with most perceiving improved sleep, which is consistent with Moyen et al. [7] and Stevenson et al. [17]. A recent systematic review concluded that personal bedding or environmental temperature regulation systems are effective in improving both sleep quality and thermal comfort [32]. The present findings partially align with this evidence, as participants in the POD group also rated thermal comfort modestly. While the perceived impact on recovery was minimal, these preliminary findings regarding satisfaction and comfort highlight the potential of temperature-controlled bedding as a noninvasive tool to aid recovery and sleep quality in high-performance sport environments.

Several limitations should be acknowledged when interpreting these findings. First, the study adopted a between-subjects design, whereby separate participant groups were allocated to each experimental condition. This design prevents direct within-subject comparisons and increases susceptibility to inter-individual variability in habitual sleep patterns and thermoregulatory responses, thereby limiting causal inference. Further, significant differences in sleep indices were present at baseline, for instance, the POD group reported consistently later wake times than the CON group at both time points, providing a greater opportunity to extend sleep duration. Further, the POD group reported significantly poorer sleep quality than the CON group at baseline, which may have influenced the magnitude of observed post-intervention changes. While sensitivity analyses adjusting for baseline values confirmed the robustness of the findings, part of the observed improvements may reflect a natural regression to the mean. Future research should aim to replicate these findings using a within-subject crossover or repeated measures design, incorporating independently validated sleep wearables with accurate sleep staging information or polysomnography to characterize the effects on sleep architecture. However, implementing randomized crossover conditions in elite athletic populations poses practical challenges due to scheduling constraints and the need to extend data collection periods beyond one month. The limited availability of mattress covers and the impracticality of asking elite athletes to sleep on the cover with the temperature regulation setting turned off made it difficult to collect within-subject control data. To mitigate this, we used the B-PSQI to retrospectively assess sleep over the two weeks before the intervention. Of note, this modified two-week recall version of the B-PSQI has not been formally psychometrically validated, which limits direct comparison with reference data based on the original one-month recall period. Additionally, biometric data were collected via the mattress system, which, while practical, may lack the accuracy of gold-standard lab-based tools. Further, ambient bedroom temperature was not measured and may have varied between participants. Indoor temperatures in Australian homes during summer are often warmer than outdoor overnight minima due to building heat retention, meaning some bedroom ambient temperatures may have fallen outside the recommended 18–22°C range. Finally, the sample included only elite male athletes from one sport, limiting the generalizability of findings to other sports and potentially to female athletes, who may respond differently to temperature manipulation interventions due to variations in core body temperature throughout the menstrual cycle [33,34].

This study identified favorable changes in subjective total sleep time, sleep quality, and B-PSQI global score associated with the use of a temperature-controlled mattress cover in elite athletes. These results align with previous research in temperature manipulation and suggest that temperature-controlled bedding may be a promising noninvasive strategy for improving perceived sleep quality, pending confirmation in randomized studies with validated objective sleep measures. These findings should be interpreted in the context of the study design and sample characteristics. Its passive, user-friendly design may make it particularly applicable during periods of intensified training, competition, or routine disruption, when athletes are most vulnerable to compromised sleep. However, the participants in the current study were not blinded to the condition they were assigned to and therefore, may have been susceptible to expectancy or placebo effects. Further, the findings of this study are preliminary and require confirmation in randomized within-subject or crossover trials within athletic populations. Therefore, future studies should incorporate appropriate within-subject controls and a more rigorous integration of subjective and objective sleep measures, using adequately powered samples, to determine whether observed benefits are attributable to the intervention itself or are influenced by external factors.

## Abbreviations

AFL: (Australian Football League)
B-PSQI: (Brief-Pittsburgh Sleep Quality Index)
bpm: (beats per minute)
CON: (control group)
HR: (heart rate)
HRV: (heart rate variability)
ICC: (intraclass correlation coefficient)
LMM: (linear mixed model)
POD: (temperature-controlled mattress cover group)
PSG: (polysomnography)
REM: (rapid eye movement)
RMSSD: (root mean square of successive differences)
SD: (standard deviation)
SE: (sleep efficiency)
SMD: (standardized mean difference)
SOL: (sleep onset latency)
T_core_: (core body temperature)
TST: (total sleep time)
TTiB: (total time in bed)

## References

[1] Fullagar HHK, Duffield R, Skorski S, et al. Sleep and recovery in team sport: current sleep-related issues facing professional team-sport athletes. Int J Sports Physiol Perform Hum Kinet Publishers Inc. 2015;10(8):950–957. doi: 10.1123/ijspp.2014-0565 25756787

[2] Mah CD, Mah KE, Kezirian EJ, et al. The effects of sleep extension on the athletic performance of collegiate basketball players. Sleep. 2011;34(7):942–950. doi: 10.5665/SLEEP.1132 PMC3119836 21731144

[3] Harding EC, Franks NP, Wisden W. The temperature dependence of sleep. Front Neurosci. 2019;13(APR):1–16. doi: 10.3389/fnins.2019.00336 31105512 PMC6491889

[4] Rogers NL, Bowes J, Lushington K, et al. Thermoregulatory changes around the time of sleep onset. Physiol Behav. 2007;90(4):643–647. doi: 10.1016/j.physbeh.2006.11.018 17197000

[5] Troynikov O, Watson CG, Nawaz N. Sleep environments and sleep physiology: A review. J Therm Biol. 2018;78(April):192–203. doi: 10.1016/j.jtherbio.2018.09.012 30509635

[6] Krauchi K, Deboer T. The interrelationship between sleep regulation and thermoregulation. Front Biosci. 2010 1; 15(1): 604–625. 10.2741/3636 20036836

[7] Moyen NE, Ediger TR, Taylor KM, et al. Sleeping for one week on a temperature-controlled mattress cover improves sleep and cardiovascular recovery. Bioengineering. 2024;11(4):352. doi: 10.3390/bioengineering11040352 38671774 PMC11048088

[8] Fullagar HHK, Vincent GE, McCullough M, et al. Sleep and sport performance. Journal of clinical neurophysiology. 2023. 10.1097/WNP.0000000000000638 36930212

[9] Walsh NP, Halson SL, Sargent C, et al. Sleep and the athlete: narrative review and 2021 expert consensus recommendations. Br J Sports Med. 2021;55(7):356–368. doi: 10.1136/bjsports-2020-102025 33144349

[10] Lan L, Pan L, Lian Z, et al. Experimental study on thermal comfort of sleeping people at different air temperatures. Build Environ. 2014;73:24–31. Internet. DOI:10.1016/j.buildenv.2013.11.024

[11] Imbergamo CM, Patankar AG, Milewski MD Current concept review sleep optimization in the young athlete [Internet].

[12] Okamoto-Mizuno K, Mizuno K. Effects of thermal environment on human sleep and thermoregulation. J Physiol Anthropol. 2012;50(4):1–9. Internet. 10.1186/1880-6805-31-14 PMC3427038 22738673

[13] Pan L, Lian Z, Lan L. Investigation of sleep quality under different temperatures based on subjective and physiological measurements. HVAC R Res. 2012;18(5):1030–1043. doi: 10.1080/10789669.2012.667037

[14] Pasquier F, Chauvineau M, Castellini G, et al. Does body cooling facilitated by bedding compared to control condition improve sleep among adults (18-64 years old)? A systematic review and meta-analysis. J Therm Biol. 2025;127(May 2024):104030. Internet. doi:10.1016/j.jtherbio.2024.104030 39708549

[15] Herberger S, Kräuchi K, Glos M, et al. Effects of sleep on a high-heat capacity mattress on sleep stages, EEG power spectra, cardiac interbeat intervals and body temperatures in healthy middle-aged men. Sleep. 2020;43(5):1–9. doi: 10.1093/sleep/zsz271 31679018

[16] Kräuchi K, Fattori E, Giordano A, et al. Sleep on a high heat capacity mattress increases conductive body heat loss and slow wave sleep. Physiol Behav. 2018;185(November 2017):23–30. Internet. doi:10.1016/j.physbeh.2017.12.014 29247670

[17] Stevenson S, Suppiah H, Mündel T, et al. Under the covers: the effect of a temperature-controlled mattress cover on sleep and perceptual measures in healthy adults. Clocks Sleep. 2025;7(4):55. doi: 10.3390/clockssleep7040055 41133665 PMC12550930

[18] McKay AKA, Stellingwerff T, Smith ES, et al. Defining training and performance caliber: a participant classification framework. Int J Sports Physiol Perform. 2022;17(2):317–331. doi: 10.1123/ijspp.2021-0451 34965513

[19] Sancho-Domingo C, Carballo JL, Coloma-Carmona A, et al. Brief version of the Pittsburgh sleep quality Index (B-PSQI) and measurement invariance across gender and age in a population-based sample. Psychol Assess. 2021;33(2):111–121. doi: 10.1037/pas0000959 33119375

[20] Haghayegh S, Khoshnevis S, Smolensky MH, et al. Novel temperature-controlled sleep system to improve sleep: a proof-of-concept study. J Sleep Res. 2022;31(6):1–12. doi: 10.1111/jsr.13662 35852479

[21] Chauvineau M, Pasquier F, Duforez F, et al. Increased training load promotes sleep propensity and slow-wave sleep in endurance runners: can a high-heat-capacity mattress topper modulate this effect? J Sleep Res. 2024;33(4):1–12. doi: 10.1111/jsr.14132 38148606

[22] Cizza G, Piaggi P, Rother KI, et al. Hawthorne effect with transient behavioral and biochemical changes in a randomized controlled sleep extension trial of chronically short-sleeping obese adults: implications for the design and interpretation of clinical studies. PLOS ONE. 2014;9(8):e104176. doi: 10.1371/journal.pone.0104176 25141012 PMC4139265

[23] Neukirch N, Colagiuri B. The placebo effect, sleep difficulty, and side effects: a balanced placebo model. J Behav Med. 2015;38(2):273–283. doi: 10.1007/s10865-014-9590-5 25119580

[24] Cao T, Lian Z, Ma S, et al. Thermal comfort and sleep quality under temperature, relative humidity and illuminance in sleep environment. J Building Eng. 2021;43(January):102575. Internet. doi:10.1016/j.jobe.2021.102575

[25] Lan L, Tsuzuki K, Liu YF, et al. Thermal environment and sleep quality: a review. Energy Build. 2017;149:101–113. Internet. DOI:10.1016/j.enbuild.2017.05.043

[26] d’Onofrio P, Jernelöv S, Rosén A, et al. The polysomnographical meaning of changed sleep quality—A study of treatment with reduced time in bed. Brain Sci. 2023;13(10):1426. doi: 10.3390/brainsci13101426 37891794 PMC10605173

[27] Chauvineau M, Hollville E, Duforez F, et al. Effect of a high-heat-capacity mattress topper on sleep in elite badminton players during a Summer training period: does 1 Size fit all? Int J Sports Physiol Perform. 2025;20(8):1152–1160. doi: 10.1123/ijspp.2024-0554 40555416

[28] Aloulou A, Leduc C, Duforez F, et al. Effect of an innovative mattress and cryotherapy on sleep after an elite rugby match. Med Sci Sports Exercise. 2020;52(12):2655–2662. doi: 10.1249/MSS.0000000000002403 32472928

[29] Sargent C, Rogalski B, Montero A, et al. The sleep behaviors of elite Australian rules footballers before and after games during an entire season. Int J Sports Physiol Perform. 2022;17(6):932–942. doi: 10.1123/ijspp.2021-0417 35100565

[30] Veale JP, Pearce AJ. Physiological responses of elite junior Australian rules footballers during match-play [Internet]. J Sports Sci Med. 2009;8(3). PMC3763274 24149992

[31] Juliff LE, Halson SL, Peiffer JJ. Understanding sleep disturbance in athletes prior to important competitions. J Sci Med Sport. 2015;18(1):13–18. Internet. doi:10.1016/j.jsams.2014.02.007 24629327

[32] Xu X, Zhang H, Schiavon S, et al. The effects of personal comfort systems on sleep: a systematic review. Renewable Sustain Energy Rev Elsevier. 2025;213:115474. doi: 10.1016/j.rser.2025.115474

[33] Baker FC, Siboza F, Fuller A. Temperature regulation in women: Effects of the menstrual cycle. Temperature. 2020;7(3):226–262. doi:10.1080/23328940.2020.1735927 PMC7575238 33123618

[34] Ferreira FC, Padilha MCSV, Tobadini E, et al. Women have a greater cardiac vagal withdrawal to heat stress compared to men. Temperature. 2022;10(4):444–453. doi: 10.1080/23328940.2022.2135354 PMC10732604 38130655

